# Food consumption frequency and perceived stress and depressive symptoms among students in three European countries

**DOI:** 10.1186/1475-2891-8-31

**Published:** 2009-07-15

**Authors:** Rafael T Mikolajczyk, Walid El Ansari, Annette E Maxwell

**Affiliations:** 1 Department of Public Health Medicine, School of Public Health, University of Bielefeld, Germany; 2 University of Gloucestershire, Faculty of Sport, Health & Social Care, Oxstalls Campus, Oxstalls Lane, Gloucester GL2 9HW, UK; 3 School of Public Health and Jonsson Comprehensive Cancer Center, University of California, Los Angeles, USA

## Abstract

**Background:**

Certain foods might be more frequently eaten under stress or when higher levels of depressive symptoms are experienced. We examined whether poor nutritional habits are associated with stress and depressive symptoms and whether the relationships differ by country and gender in a sample from three European countries collected as part of a Cross National Student Health Survey.

**Methods:**

A cross-sectional survey was conducted among first-year students in Germany (N = 696), Poland (N = 489) and Bulgaria (N = 654). Self-administered questionnaires included a 12-item food frequency questionnaire, Cohen's Perceived Stress Scale, and a modified Beck Depression Index. Linear regression analyses were conducted for two outcomes, perceived stress and depressive symptoms.

**Results:**

Food consumption frequencies differed by country and gender, as did depressive symptoms and perceived stress. For male students, none of the food consumption groups were associated with perceived stress or depressive symptoms. In females, perceived stress was associated with more frequent consumption of sweets/fast foods and less frequent consumption of fruits/vegetables. Additionally, depressive symptoms were associated with less frequent consumption of fruits/vegetables and meat.

**Conclusion:**

Our data show consistent associations between unhealthy food consumption and depressive symptoms and perceived stress among female students from three European countries, but not among male students. This suggests that efforts to reduce depressive symptoms and stress among female students may also lead to the consumption of healthier foods and/or vice-versa.

## Introduction

Dietary habits are a major aspect of people's lifestyles that influence health, morbidity, and mortality for a range of conditions. Hence, patterns of food consumption and their relation to mental health have received some attention in research [1]. For example, some observational and experimental studies explored the effects of carbohydrate intake on mood [2-4]. Other studies have assessed the association between stress and food selection, reporting partly conflicting results [5-11]. Carbohydrate consumption has been hypothesized to relieve depressive moods [12], and this has been considered as part of the causal link for developing obesity [13,14]. However, the association has also been seen in the opposite direction, with stress and depressive symptoms resulting in poorer food choices [15]. Indeed, studies on the effects of stress on food choice show that people experiencing periods of stress reported overeating foods they would normally avoid, and that they ate these foods to feel better [16].

In children, stress was correlated with being overweight [17,18], unhealthy eating behaviours, as well as with the use of eating as a coping mechanism [19]. For adolescents of both genders in the U.S., the lack of a healthy diet was associated with reporting depression/stress for 10 or more days in the past month [20]. Stress not only increased food consumption in certain individuals but also shifted their food choices from lower fat to higher fat foods [16].

Whilst eating has been theorized as a coping strategy for stressful situations [19], less is known about the association of stress or depression with the frequency of consumption of various food groups among college students [15]. For instance in Germany, students preparing for exams had higher stress and more tendency to eat in order to distract themselves from stress than compared to control students [21]. In the U.S., weight gain in college women was negatively associated with students being stress-free, eating vegetables, and consuming less high-cholesterol foods [22]. Additionally, given that 65% of United Arab Emirates university students reported that their stress levels were too high and 50% reported that their diet was unhealthy [23], the importance of understanding students' patterns of consumption of food groups becomes evident. If poor nutritional habits are associated with stress and/or depressive symptoms, programs addressing mental health may also lead to the consumption of healthier foods and/or vice-versa.

Although depression is often related to appetite changes [24], little research has been done to examine the relationship between healthy eating and depressed mood [25]. In fact, until recently, no studies had examined the relationship between depression and nutritional intake, e.g., among adolescents [25]. Previous studies that examined this link usually focused on single countries, e.g., the U.S. [25,26], the UK [27], or China [15]. When single countries are examined, participants usually share the same underlying nutritional habits and other background characteristics that are potentially related to perceived stress or depressive symptoms. Single-country studies are also rarely directly comparable to one another due to the differences in measures of food consumption or mental health indicators. This has led to suggestions that future research on nutrition and on the correlation between depression and food consumption should be conducted across diverse student populations [15]. University students are particularly important as they have greater freedom and control over their lifestyles, and health behaviours formed during young adulthood may have sustaining impacts on health throughout later life [28]. Hence, it is of interest to assess the extent of association between indicators of mental health (such as perceived stress and depressive symptoms) and nutritional habits.

The aim of the analysis described in this paper is to assess the associations between mental health and nutritional habits across culturally diverse samples of students from three European countries. The analysis explores the links between reported levels of perceived stress and depressive symptoms and the consumption of selected food groups. The three samples consisted solely of first-year university students in each of the three countries; hence, sources of variability, such as education level or socio-economic status, are reduced.

## Methods

### Sample

The dataset used in this analysis is part of the Cross National Student Health Survey (CNSHS) [29], a general health survey among student populations conducted in several European countries. The current dataset is from three universities in three countries: University of Bielefeld, Germany (DE); Catholic University of Lublin, Poland (PL); and Sofia University, Bulgaria (BG). Papers related to depressive symptoms in these three countries have already been published [30,31]. The questionnaire was initially developed in German and then translated into Polish and Bulgarian using two independent translations for each language. Cases of disagreement were reviewed by the research team and decisions were made by the authors familiar with the respective languages (usually native speakers). Approval of the study and data collection at the three participating universities took place in 2005. The participating universities were selected based on the first author's appointment (University of Bielefeld) and working relationships with colleagues (Catholic University of Lublin; Sofia University). The protocol was the same across the three countries: first-year undergraduate students were invited to complete a self-administered questionnaire towards the end of a course lecture. The courses were selected to achieve a locally representative sample that was also comparable between the three sites, with one-third of students from each university coming from each of the three discipline areas: natural sciences, social sciences and language, and law and economy. Within each discipline area, courses were randomly selected until the expected quota was reached. We had planned to recruit about 500 students at each institution, but due to some large courses we exceeded this goal in Germany and Bulgaria. Students were informed about the study aims and that by completing a questionnaire they provided their informed consent to participate in the study. Participation was voluntary and anonymous, and withdrawal from the study was possible at any stage. Response rates varied among the sites: 85% in Germany and over 95% in both Poland and Bulgaria. The total number of respondents from the three sites was 2,103 students, although the current analysis is based on 1,839 (87%) respondents due to some missing values.

### Variables

#### Assessment of dietary intake

Students completed a food frequency questionnaire (12 indicator variables) that measured their consumption of sweets, cakes/cookies, snacks and fast/canned food, fresh fruits, raw and cooked vegetables and salads, meat and fish, milk products, and cereals (see Table 1). The instrument was created to include food groups that are important when studying dietary habits, in line with other research studies [32]. The introductory question, "How often do you eat the following foods?" asked participants about the frequency of their usual consumption of each food group separately (rated on a 5-point scale: several times a day, daily, several times a week, 1–4 times a month, and never). Both the face and content validity of the instrument were ascertained by grounding the questionnaire on literature review. No formal test of validity was performed, but the questionnaire was very similar to other food frequency questionnaires that had been validated, e.g., [33,34].

**Table 1:** Food consumption and mental health indicators by country and gender

	DE		PL		BG	
	Female	Male	Female	Male	Female	Male
	N = 394	N = 302	N = 351	N = 138	N = 449	N = 205
Food Group^a^	Mean (SD)	Mean (SD)	Mean (SD)	Mean (SD)	Mean (SD)	Mean (SD)
Sweets	3.3 (1.0)	3.0 (1.0)	3.1 (0.9)	3.1 (0.8)	3.6 (0.9)	3.4 (0.9)
Cakes/cookies	2.3 (0.6)	2.2 (0.6)	2.8 (0.8)	2.7 (0.8)	3.1 (0.9)	3.0 (0.9)
Snacks	2.1 (0.7)	2.3 (0.7)	2.1 (0.8)	2.2 (0.9)	2.8 (0.9)	2.7 (0.8)
Fast food	2.2 (0.6)	2.4 (0.6)	1.8 (0.7)	2.0 (0.8)	3.1 (1.0)	3.5 (0.9)
Fresh fruits	3.5 (0.9)	3.2 (0.9)	3.3 (0.9)	3.0 (0.9)	3.6 (0.9)	3.3 (0.8)
Salad	3.3 (0.8)	3.1 (0.8)	3.1 (0.8)	3.0 (0.8)	3.7 (0.9)	3.4 (0.8)
Cooked vegetables	3.1 (0.8)	3.0 (0.8)	2.7 (0.9)	2.6 (0.9)	3.0 (0.9)	3.1 (0.9)
Soft drinks	2.5 (1.1)	2.9 (1.0)	2.9 (1.1)	3.1 (0.9)	2.9 (1.2)	3.3 (1.1)
Meat	3.0 (1.0)	3.6 (0.9)	3.3 (0.9)	3.6 (0.9)	3.1 (1.0)	3.7 (0.9)
Fish	2.0 (0.7)	2.2 (0.8)	2.2 (0.7)	2.3 (0.7)	2.3 (0.7)	2.5 (0.8)
Milk products	3.9 (0.9)	3.9 (0.9)	3.3 (1.0)	3.2 (1.0)	3.8 (0.8)	3.7 (0.9)
Cereal/cereal products	3.9 (0.9)	3.6 (1.1)	3.6 (1.0)	3.5 (1.1)	3.5 (1.1)	3.8 (1.1)
Mental Health Indicators						
M-BDI^b^	28.1 (15.6)	25.2 (14.7)	33.6 (17.4)	27.6 (15.2)	33.6 (15.4)	29.9 (13.6)
PSS^c^	25.2 (8.1)	22.9 (8.0)	29.3 (7.5)	25.0 (6.9)	26.5 (7.8)	23.8 (7.3)

^a ^Mean of the consumption frequency scale from 1 = never to 5 = several times per day

^b ^Mean Modified Beck Depression Index, higher scores indicate stronger depressive symptoms

^c ^Mean Perceived Stress Scale by Cohen, higher scores indicate higher perceived stress

#### Stress and depressive symptoms measures

Perceived stress was measured with Cohen's Perceived Stress Scale (PSS -14 items) [35], which assesses the extent to which a respondent considers life situations to be stressful. The questions measure how unpredictable, uncontrollable, and overloaded respondents find their lives, using a 5-point Likert scale response format ('0 = Never', '4 = Very Often'). Scores for individual participants were obtained by summing their responses to all 14 items. Employing the PSS scale in a population probability sample in the U.S., Cohen & Williamson [36] reported an internal reliability (Cronbach's alpha) of 0.78. The scale's predictive and discriminant validities were also established in studies that linked the measured concept of stress to health outcomes [36,37]. In our sample, Cronbach's alpha of the PSS was 0.85 in Germany, 0.81 in Poland, and 0.80 in Bulgaria [31].

Depressive symptoms were measured using a modification of the Beck Depression Inventory (M-BDI) [38,39]. The modification of the original BDI included two approaches: (a) the four items per symptom, which assessed the specific symptom's intensity in the original BDI, were replaced by a single statement per symptom with a six-point Likert scale measuring its frequency in the last 4 weeks (with the two extreme categories labelled as 0 = 'Never', 5 = 'Almost Always'); and (b) one symptom, which exhibited low specificity (loss of weight) was excluded. The reduction in the number of items per symptom is consistent with another recent modification of the BDI (BDI-II [40]). The German language M-BDI, along with other versions of the BDI, computes a single score for individual respondents by summing their responses for all items of the scale. Through a German general population sample and in selected subsamples [38,39], the authors of the M-BDI have demonstrated its construct validity and measurement equivalence as compared to the original BDI. The authors have also provided a cut-off score for screening for clinically relevant depressive symptoms at ≥ 35, corresponding to the 85th percentile of the representative sample of the German population [41]. In our sample, Cronbach's alpha of M-BDI was 0.90 in Germany, 0.92 in Poland, and 0.87 in Bulgaria [31].

### Statistical analysis

The analysis was conducted using SPSS 13, with statistical significance level set at p < 0.05. We present descriptive data on food consumption and mental health indicators (means and standard deviations of the item values) by country and gender. We assessed univariable associations between consumption of food groups and both mental health indicators using linear regression stratified by gender and adjusted for country. In order to reduce correlations between consumption of similar food groups, we built subscales for some food groups prior to adjusting for all food groups in the multivariable regression. Based on theoretical considerations regarding the content of foods, carbohydrate dense foods (sweets, cake/cookies, snacks, and fast food) were combined in one subscale; similarly, one subscale was created for fresh fruits and salads and cooked vegetables. This approach was supported by at least moderate correlations (Spearman rho >0.2) among the items of each subscale and factor analysis (results not shown). The subscales were created as mean scores of the corresponding items. The two new subscales and the remaining individual food groups were tested for multicollinearity with respect to the outcome variables. All variables displayed variance inflation factors far below 5, which could be an indication of serious multicollinearity problems [42]. The independent associations between food groups and perceived stress and depressive symptoms were studied in two separate multivariable linear regression models. Given the gender and country differences across depressive symptoms, the analysis was stratified by gender and adjusted for country. Additionally, we included interaction terms to test whether the associations between specific food groups and mental health indicators differed across countries.

## Results

### Characteristics of the sample

Table 1 shows that 65.3% of the study sample was female. The country distribution of the students was 38% from Germany, 27% from Poland, and 35% from Bulgaria. Participants' average age was 20.6 years (SD = 2.3 years). About 2% of students in Germany, 22% in Bulgaria, and 56% in Poland were <20 years, with a very narrow age range in Poland and Bulgaria (data not shown).

### Food consumption behaviour and mental health indicators

The frequency of food consumption differed by country and gender (Table 1). Consumption of sweets, cakes, snacks, and fast food was generally more common in Bulgaria than in Poland or Germany. In all countries, the consumption of sweets and cakes was more common in females than in males, and conversely, the consumption of fast foods was more common in males. Eating fresh fruits, salads and cooked vegetables, milk products, and cereals was only slightly more common in females, while the consumption of soft drinks/lemonade, meat, and fish was more common in males. Females displayed higher scores of depressive symptoms and perceived stress than males. There were also differences in depressive symptoms across countries.

### Associations between food intake and perceived stress or depressive symptoms

The univariable associations are presented in Table 2. The similar associations between the consumption of sweets, cakes, snacks, and fast food and mental health indicators, and between the consumption of fruits, salads and vegetables and mental health indicators support our decision to combine these food groups into subscales. Because the association between the consumption of fast food and mental health indicators deviated from this pattern among male students, we conducted a more detailed analysis stratified by country and found that the effect existed only in Poland, while no significant associations were seen in Bulgaria and Germany.

**Table 2:** Associations between food consumption and perceived stress and depressive symptoms (each food group adjusted solely for country, separate models for males and females and for both mental health indicators)

	Perceived Stress score (PSS)				Depressive Symptoms score (M-BDI)			
	Female		Male		Female		Male	
**Food group**	p-value	Estimate*	p-value	Estimate*	p-value	Estimate*	p-value	Estimate*
Sweets	0.04	0.54	0.37	-0.31	0.27	0.59	0.10	-1.11
Cookies	0.13	0.48	0.46	-0.30	0.79	0.17	0.27	-0.89
Snacks	0.04	0.66	0.92	0.04	0.33	0.62	0.34	-0.76
Fast food	0.07	0.55	0.04	0.84	0.57	0.34	0.02	1.85
Fruits	<0.001	-1.17	0.37	-0.32	0.002	-1.69	0.53	-0.45
Salads	<0.001	-1.21	0.23	-0.48	<0.001	-2.55	0.25	-0.88
Vegetables	0.003	-0.82	0.97	-0.01	0.003	-1.62	0.29	0.77
Soft drinks	0.52	-0.14	0.60	0.17	0.05	-0.90	0.55	-0.36
Meat	0.03	-0.52	0.80	0.09	0.003	-1.47	0.24	-0.78
Fish	<0.001	-1.32	0.75	-0.13	0.02	-1.60	0.90	0.10
Milk products	0.009	-0.72	0.34	-0.34	0.06	-1.07	0.08	-1.18
Cereal products	0.17	-0.33	0.17	-0.41	0.17	-0.67	0.07	-1.02

* Change in the corresponding score (PSS or M-BDI) per one unit of the food group frequency scale

In the multivariable analysis stratified by gender, the two-way interactions between countries and food groups were not statistically significant for either perceived stress or depressive symptoms, indicating a similar relationship between food intake and stress and between food intake and depressive symptoms. A single exception was an interaction between country and fish consumption with respect to depressive symptoms in females, with higher levels of depressive symptoms in females consuming more fish in Germany, lower levels of depressive symptoms in females consuming more fish in Bulgaria, and no association in Poland. Subsequently, the interaction terms were removed from both models and the country variable was retained in the analysis to adjust for confounding.

For male students, none of the food consumption subscales or food groups were associated with perceived stress or depressive symptoms (Table 3). In females, higher consumption of carbohydrate dense foods such as sweets, cookies, snacks, and fast food was associated with higher perceived stress, but not with depressive symptoms scores. A less frequent consumption of fruits and vegetables was associated with both higher perceived stress and depressive symptoms scores. (The partial eta square was nearly equal in both models, data not shown). Additionally, there was a negative association between meat consumption and depressive symptoms among females.

**Table 3:** Associations between food consumption and perceived stress and depressive symptoms (multivariable analysis adjusted for country and for all other variables in the table, separate models for males and females and for both mental health indicators)

	Perceived Stress score (PSS)				Depressive Symptoms score (M-BDI)			
	Female		Male		Female		Male	
**Food group or subscale**	p-value	Estimate*	p-value	Estimate*	p-value	Estimate*	p-value	Estimate*
Sweets/Cookies/Snacks/Fast food^#^	0.03	0.72	0.29	-0.46	0.15	0.96	0.30	-0.89
Fruits/Vegetables^+^	<0.01	-1.17	0.43	-0.40	<0.01	-2.37	0.94	-0.07
Soft drinks	0.83	-0.05	0.23	0.40	0.16	-0.64	0.64	0.29
Meat	0.11	-0.40	0.91	0.04	0.01	-1.38	0.34	-0.66
Fish	0.06	-0.69	0.83	-0.09	0.95	-0.05	0.71	0.31
Milk products	0.12	-0.44	0.49	-0.26	0.23	-0.72	0.22	-0.87
Cereal products	0.99	0.00	0.38	-0.28	0.61	-0.26	0.17	-0.79

* Change in the corresponding score (PSS or M-BDI) per one unit of the food group frequency scale

** Sweets/cookies/Snacks/Fast food subscale: mean of four items (sweets, cakes/cookies, snacks, fast food)

^+ ^Fruits/Vegetables subscale: mean of three items (fresh fruits, salads, cooked vegetables)

## Discussion

Today's college students are tomorrow's parents, role models, and patients. Unhealthy food practices and habits could impose health risks later in life and could be passed on to the next generation. Diet has an effect on mood [43], and although the emotional well being of university students has been studied [44], recent reports indicate that depressive symptoms in students are of principal importance and require further study [45]. Our findings show that the relationships between food consumption and depressive symptoms and perceived stress were consistent across the studied countries but different by gender. While in males there was no association between consumption of food groups and the above-mentioned mental health indicators, the associations were quite strong in female students.

We found associations between the consumption of sweets/fast food and fruits/vegetables and perceived stress and between the consumption of fruits/vegetables and meat and depressive symptoms. The association between higher consumption of sweets and higher perceived stress levels was also reported by others: sweets, chocolate, cake, and biscuits were eaten more often under stress [8,46]. Food composition (e.g., high fat/energy content) may influence people's selections of certain foods during life events that are stressful [46]). On the other hand, stress might trigger a loss of control mechanisms by which people avert themselves from consuming perceived fattening or unhealthy foods [16]. That "meal-type" foods, which often consist of salads or vegetables, are also eaten less frequently during periods of stress [8] might explain the observed negative correlation between fruits/vegetables and perceived stress.

Higher consumption of fruits/vegetables was associated lower levels of depressive symptoms among females in our study. The direction of this association seems to be due to the behavioural consequences of higher depressive symptoms and is consistent with the correlation between depressive symptoms and perceived stress [31]. Interestingly, more frequent meat consumption was associated with lower levels of depressive symptoms in females. The interpretation of this finding is not entirely clear. Possibly, again the consumption of meat indicates better mood, rather than that meat positively affects mood.

Conversely, fish consumption (rich in omega-3 fatty acids) might be causally associated with mood stabilization [47], with some evidence that levels of these fatty acids are linked to depression [48]. However, we did not find any association between fish consumption and depressive symptoms, consistent with Hakkarainen et al [49] who reported that dietary intake of omega-3 fatty acids showed no association with low mood level. Indeed, the evidence base on the relationships between fish consumption and depressive symptoms remains inconclusive. For instance Hakkarainen et al [50] found that subjects reporting anxiety or depressed mood had *higher *intake of omega-3 and omega-6 fatty acids – findings that conflict with previous reports of the beneficial effects of these fatty acids on mood, where lower prevalence of depressive symptoms was reported among people consuming high amounts of fish [51,52]. These conflicting findings probably reflect the different dietary intake levels of omega-3 fatty acids employed in different trials (ranging from 9.6 g/day, [47] to 2.2 g/day, [49]), and the lack of distinction between consumption of white versus fatty fish, which have varying contents of fatty acids (0.48 g/100 g in white fish (cod) to 5.33 g/100 g in fatty fish (mackerel) [53].

While an increased consumption of sweets/fast food was associated with higher levels of perceived stress, the absence of this correlation for depressive symptoms might indicate that increased consumption partly relieves depressive symptoms. Carbohydrate ingestion has been hypothesized to relieve depressive moods [12], but such effects could be mediated through psychological factors rather than nutrient content of sweets [54]. Hence future studies of the influence of carbohydrates on mood will need to consider people's pre-existing psychological and physiological functioning [2].

All the above findings were observed only in women. In males, none of the associations were significant and most of the estimates were closer to zero. The single exception was increased stress and depressive symptoms in Polish males with a more frequent fast food consumption observed in univariable analysis. Since the effect was restricted solely to Polish male students and not seen for male students in the other countries, and we are not aware of any theoretical justification, we see this as a chance finding. The association disappeared in multivariable analysis using the subscale for carbohydrate dense foods. Previous studies assessing the association of food intake with one or both mental health indicators in Chinese [15] and American [15] populations did not investigate the potential effect modification by gender, therefore the global associations they observed might have also resulted from associations limited to women only. Many individual, social, and political factors influence food selection and eating patterns [55]. Hence, gender differences may result from different responses to perceived stress with respect to food consumption: either increased (hyperphagia) or decreased (hypophagia) and different prevalences of these responses by gender [8]. Females, being more often restrained eaters [56], might react more strongly to perceived stress and/or depressive symptoms in their food intake. The preparation of healthy items (salads and cooked vegetables) might also require some additional efforts. Therefore under stress or decreased motivation due to depressive symptoms their consumption might decrease. Other explanations include female perceptions of self- and body-image and self-esteem, which have been associated with depression and might be associated with food consumption [57,58].

### Strengths and limitations

In all three countries, data were collected by the same method, and the treatment of the data was the same across sites. Since there may be regional variations and systematic differences between universities in each country, the data from single universities may not be representative for the countries included in this analysis. However, a high response rate at each site guaranteed that selection bias was not a likely threat to the internal validity of the study. Both mental health indicators and food consumption assessment were based on self-report and may be subject to social desirability bias. Furthermore, food frequency questionnaires do not assess serving sizes, which increases unmeasured variation, decreases precision, and possibly also affects validity of the measurement. When combining food groups into subscales, we assumed that students who eat only salads, for example, do not eat larger portions than students who eat salads, fruits, and vegetables. The food frequency questionnaire used in this study was not tested against objective and more sophisticated methods of food consumption measurement. Nevertheless, the instrument was similar to other food frequency questionnaires for which some proof of validity exist [33,34]. A further specific limitation is that we did not ask whether students ate at the university canteen/refectory or at their parental home. In both these cases, students may have less control over what they eat, especially at meal times.

Finally, given the cross-sectional nature of the study, we were not able to assess the direction of the association between food consumption and mental health. Given the absence of physiological mechanisms by which fruits or vegetables diminish perceived stress or depressive symptoms, it is most likely that the observed negative association resulted as effects of mental health on food consumption.

## Conclusion

Using data from three European countries from a study with a common methodology, we showed an association between mental health indicators (perceived stress and depressive symptoms) and food intake in female, but not male, students. Our findings suggest that interventions oriented towards perceived stress and depressive symptoms in female students should also address the issue of healthy nutrition. Additionally, efforts to reduce depressive symptoms and stress among female students may lead to the consumption of healthier foods and/or vice-versa.

## Competing interests

The authors declare that they have no competing interests.

## Authors' contributions

RTM designed the research question, conducted the analysis and drafted the results and methods sections. WEL contributed to the research question and wrote the final manuscript. AEM participated in writing the manuscript.
